# Differences in call profiles, interventions and physician staffing between ground and helicopter emergency medical services in Austria: A sub-analysis of the Austrian Emergency Day 2024 audit

**DOI:** 10.1007/s00508-025-02684-7

**Published:** 2026-01-08

**Authors:** Romana Erblich, Martin W. Dünser, Christian Anzur, Stefan Dressler-Stross, Wolfgang Voelckel, Mario Krammel, Adolf Schinnerl, Berndt Schreiner, Helmut Trimmel

**Affiliations:** 1 Department of Anaesthesiology and Intensive Care Medicine, Kepler University Hospital and Johannes Kepler University, Krankenhausstraße 9, 4020 Linz, Austria; 2 Institute for Mathematics and Statistics, Faculty of Engineering, Fachhochschule Wiener Neustadt, Wiener Neustadt, Austria; 3 Institute for Scientific Methods and Market Research, Faculty of Economics, Fachhochschule Wiener Neustadt, Wiener Neustadt, Austria; 4 Department of Anaesthesiology and Critical Care Medicine, AUVA Trauma Center Salzburg, Academic Teaching Hospital of the Paracelsus Private Medical University, Salzburg, Austria; 5 Emergency Medical Service Vienna, Vienna, Austria; 6 Team Ärztlicher Rettungsdienst, Land Tirol, Innsbruck, Austria; 7 Red Cross Lower Austria, Tulln, Austria; 8 Department of Anaesthesia, Emergency and General Intensive Care Medicine, University Hospital Wiener Neustadt, Wiener Neustadt, Austria; 9 Karl Landsteiner Institute for Emergency Medicine, Wiener Neustadt, Austria

**Keywords:** HEMS, EMS, Severity, NACA, Interfacility transfer

## Abstract

**Objective:**

To compare the call profiles, physician-delivered interventions and physician staffing of ground emergency medical services (GEMS) and helicopter emergency medical services (HEMS) in Austria.

**Methods:**

This was a sub-analysis of the Austrian Emergency Day 2024 audit, which was a prospective, observational, nationwide study conducted across 98 out of 149 public physician-staffed emergency medical services (EMS) in Austria. During 24 hours all emergency calls, to which EMS physicians were dispatched, were documented. Data collection included characteristics of participating EMS bases and non-patient-related details of each emergency call.

**Results:**

Included in the study were 79 GEMS bases conducting 338 calls and 19 HEMS bases conducting 60 calls were included. The proportion of primary calls was lower (71.7% vs. 83.7%) and the proportion of interfacility transfers higher (18.3% vs. 4.7%) in HEMS than GEMS (*p* < 0.001). The median (interquartile range, IQR) duration of emergency calls was longer for HEMS than GEMS missions with 62min (IQR 53–71min) vs. 47min (IQR 44–50min) (*p* < 0.001). The National Advisory Committee for Aeronautics (NACA) score was lower for GEMS than HEMS calls (3 (2–4) vs. 4 (3–5); *p* < 0.001). The over-triage rate was higher in the GEMS than HEMS group (58.4% vs. 29.6%; *p* < 0.001). Except for sonography use, no difference in the rate of diagnostic or therapeutic physician-delivered interventions was observed between GEMS and HEMS. Characteristics of physician staffing did not differ between GEMS and HEMS.

**Conclusion:**

Relevant differences in the call profiles but not physician-delivered interventions or physician staffing exist between GEMS and HEMS in Austria. The HEMS are more frequently tasked to emergencies with a higher severity and conduct interfacility transfers more frequently than GEMS.

## Introduction

Prehospital emergency medical services (EMS) in Austria operate as a two-tiered model consisting of ambulances staffed by non-physician personnel, BLS-providers (Basic Life Support; Rettungssanitäter:innen) or ALS-providers (Advanced Life Support; Notfallsanitäter:innen) and physician-staffed rapid response units [1, 2]. As each federal state of Austria organizes local EMS, system design varies across Austria. While some states operate ALS ambulances and dispatch physician response units only to apparently life-threatening events, others work with BLS ambulances only and dispatch EMS physicians more liberally [1, 2]. The training of EMS physicians in Austria comprises anesthesiological, medical, surgical and critical care competencies based on a legally defined theoretical and clinical training curriculum, requiring at least 33 months of postgraduate clinical experience [3]. Comparative studies indicate that Austria, along with Germany, has one of the highest rates of prehospital EMS physician involvement in Europe [4, 5].

In Austria, physician-staffed EMS operate by ground (e.g., using rapid response cars; ground EMS, GEMS) or air (e.g., using helicopters; helicopter EMS, HEMS) [1, 4–7]. For 9 million inhabitants, 149 physician-staffed EMS (GEMS, *n* = 119; HEMS, *n* = 30) are tasked by public emergency control centers. In response to tourism-related increases in population numbers during winter months, the number of physician-staffed EMS is increased to 124 GEMS and 43 HEMS from November until April [8]. While physician-staffed GEMS are based both in urban and rural settings providing services 24 h per day, the operational radius of HEMS is greater also covering remote and mountainous regions [6, 7]. The majority of Austrian HEMS operate only during prolonged daylight hours. So far, little is known about differences in the emergency medical spectrum and physician staffing between GEMS and HEMS in Austria.

This explorative study compares call profiles, physician-delivered interventions, and physician staffing of 79 GEMS and 19 HEMS in Austria.

## Material and methods

The study was designed as a sub-analysis of the Austrian Emergency Day 2024 audit, which was a prospective, observational, nationwide study conducted across 98 out of 149 public physician-staffed EMS in Austria on 3 October 2024. During 24 h starting on the audit day at 6.00 am, all emergency calls, to which EMS physicians were dispatched, were documented using an online case report form. Data collection included characteristics of participating EMS bases as well as non-patient-related details of each emergency call conducted during the 24 h audit period. Both the protocol of the audit [8] and its main results [9] have previously been published. The study protocol was evaluated by the Ethics Committee of Lower Austria (GS3-EK-12/808-2024). As participation was voluntary and no patient-related data were collected, formal approval or written informed consent was waived. The Austrian Emergency Day 2024 audit was funded by the Karl Landsteiner Institute for Emergency Medicine located in Wiener Neustadt. The funder had no role in the study design, data collection, interpretation of the study results, drafting or approval of the manuscript. The present manuscript was prepared according to the updated STROBE checklist for reporting cohort studies [10].

### Study variables

For the present analysis, the following variables were extracted from the two databases of the Austrian Emergency Day 2024 audit: 1) database including details of the participating physician-staffed EMS: operational type (GEMS, HEMS), Austrian state where the EMS was based, number of physicians per EMS base as well as sex, age, specialty status and experience in prehospital emergency medicine (expressed by the number of active years as an EMS physician) of the EMS physician on call on the audit day. 2) Database including details of each emergency call: type of emergency call (primary, interfacility transfer, cancellation), type of dispatch (immediate dispatch, crew request), medical category of emergency call, type and number of diagnostic and therapeutic interventions delivered by the EMS physician, patient transportation mode, place of patient handover in the hospital (in case patient transport was accompanied by the EMS physician), the National Advisory Committee for Aeronautics (NACA) score [11] and duration of the emergency call.

### Definitions

As physician-staffed EMS are intended to be dispatched to medical emergencies which are potentially life-threatening (NACA score 4) or life-threatening (NACA score 5 or higher), we considered dispatch of a physician-staffed EMS to an emergency case, the severity of which was subsequently graded with a NACA score lower than 4 as over-triage. As cancellations of physician-staffed EMS have a variety of reasons, we did not include the cancelled calls into the calculation of over-triage rates. The following diagnostic and therapeutic interventions were considered to be deliverable only by an EMS physician: shock therapy, rapid sequence induction and emergency anesthesia, invasive ventilation, advanced cardiac life support, reduction of joint/fractures under periprocedural sedation, thoracostomy or chest tube insertion, cardioversion, pacemaker therapy, management of obstetric and perinatal emergencies, invasive arterial blood pressure monitoring, blood gas analysis, palliative care, sonography and verification of death in the absence of obvious clinical signs of death.

### Study objectives

The primary objective of this study was to compare the profiles of and physician-delivered interventions during emergency calls between GEMS and HEMS. The secondary objective was to compare characteristics of EMS physicians staffing GEMS and HEMS.

### Statistical analysis

All statistical analyses were conducted using the SPSS software program (IBM SPSS Statistics 30.0.0.0; IBM, Armonk, NY, USA). No imputation methods were used to compensate for missing data. Shapiro-Wilk tests were applied to evaluate whether continuous variables were normally distributed but this was not the case for any parameter. To fulfil both primary and secondary study objectives, categorical and continuous variables were compared between GEMS and HEMS using χ^2^ and Mann-Whitney U tests, respectively. A *p*-value < 0.05 was considered to indicate statistical significance. Given the explorative character of our analysis, we did not adjust alpha levels for multiple comparisons. Accordingly, *p*-values should be interpreted descriptively rather than confirmatively. Continuous study variables were presented as median values with interquartile ranges (IQR), while categorical variables were given as absolute numbers with percentages.

## Results

**Fig. 1:**
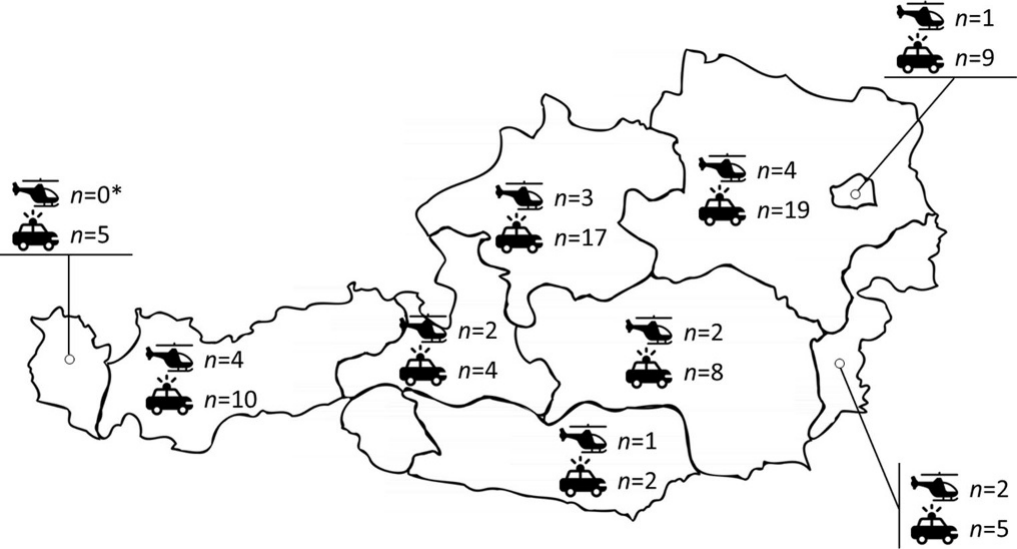
Map of Austria showing the number of participating ground and helicopter emergency medical services in the nine Austrian states. *No HEMS base from the state of Vorarlberg was included into the analysis of the Austrian Emergency Day 2024, as no HEMS call was conducted during the 24 h audit period. Map by vecteezy.com; icons by flaticon.com

In total 98 of 149 (65.8%) physician-staffed EMS participated in the Austrian Emergency Day 2024 audit and reported at least 1 emergency call on the audit day. Of the 98 physician-staffed EMS, 79 were GEMS (80.6%) while 19 were HEMS (19.4%) bases. Except for HEMS data from the state of Vorarlberg where no HEMS call was conducted during the 24 h audit period, GEMS and HEMS data were available from all other Austrian states (Fig. 1). In total, 338 GEMS (4.2, 3.5–4.8 calls per GEMS base) and 60 HEMS (2.4, 1.8–3.2 calls per HEMS base) calls were analyzed. This corresponded to 82.4% (398/483) of all emergency calls conducted by physician-staffed EMS in Austria on the audit day.

### Call profiles and physician-delivered interventions

**Fig. 2:**
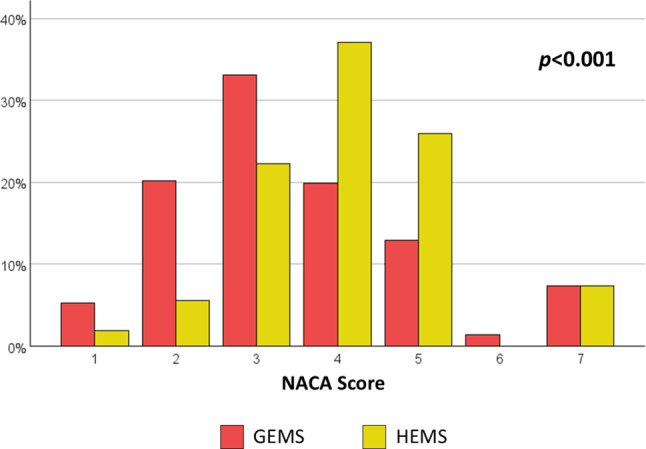
Distribution of NACA scores across GEMS and HEMS calls. *GEMS* ground emergency medical services, *HEMS* helicopter emergency medical services, *NACA* National Advisory Committee for Aeronautics

The proportion of primary calls was lower and the proportion of interfacility transfers higher in the HEMS than the GEMS group. The HEMS physicians accompanied patients during transportation to the hospital more often than GEMS physicians. Emergency patients transported by HEMS were more frequently handed over in the resuscitation room, intensive care unit or other hospital sites than patients transported by GEMS. The duration of emergency calls was longer for HEMS than GEMS missions (Table 1). The NACA scores differed between GEMS and HEMS calls (Fig. 2). The median (IQR) NACA score was lower for GEMS than HEMS calls (3 (2–4) vs. 4 (3–5); *p* < 0.001). Excluding cancelled calls, the rate of over-triage was higher in GEMS than HEMS (171/293 (58.4%) vs. 16/54 (29.6%); *p* < 0.001). Sonography was used more often by HEMS than GEMS physicians. No difference in the rate of other diagnostic or therapeutic physician-delivered interventions was observed between GEMS and HEMS (Table 2).

**Table 1:** Call profiles of physician-staffed ground and helicopter emergency medical services

		GEMS	HEMS	*p*-value
*N*		338	60	
**Type of call**	*n* (%)			< 0.001*
*Primary*		283 (83.7%)	43 (71.7%)	
*Interfacility transfer*		16 (4.7%)	11 (18.3%)	
*Cancellation*		39 (11.5%)	6 (10.0%)	
**Type of dispatch**	*n* (%)			0.72
*Immediate dispatch*		258 (76.3%)	49 (81.7%)	
*Crew request*		80 (23.7%)	11 (18.3%)	
**Category of emergency call**	*n* (%)			0.08
*Medical*		156 (46.2%)	28 (46.7%)	
*Trauma*		45 (13.3%)	14 (23.3%)	
*Neurological*		29 (8.6%)	9 (15.0%)	
*Pediatric (including trauma)*		19 (5.6%)	1 (1.7%)	
*Other*		53 (15.7%)	3 (5.0%)	
*Missing*		36 (10.7%)	5 (8.3%)	
**Patient transportation mode**	*n* (%)			0.02*
*Transport accompanied by EMS physician*		139 (41.1%)	37 (61.7%)	
*Transport unaccompanied by EMS physician*		117 (34.6%)	13 (21.7%)	
*No transport*		28 (8.3%)	1 (1.7%)	
*Death at scene*		18 (5.3%)	4 (6.7%)	
*Missing*		36 (10.7%)	5 (8.3%)	
**Place of patient handover in hospital**	*n* (%)			< 0.001*
*Emergency department*		86 (61.9%)	12 (32.4%)	
*Resuscitation room*		20 (14.4%)	8 (21.6%)	
*ICU*		19 (13.7%)	4 (10.8%)	
*Other*^*$*^		14 (10.1%)	13 (35.1%)	
**Duration of call**	*min*	47 (44–50)	62 (53–71)	< 0.001*

*GEMS* ground emergency medical service, *HEMS* helicopter emergency medical service, *ICU* intensive care unit

^$^ Coronary angiography suite, neuroradiology department, operating room, etc.

* Significant group difference

**Table 2:** Physician-delivered interventions delivered during emergency calls in physician-staffed ground and helicopter emergency medical services

		GEMS	HEMS	*p*-value
*n*		338	60	
**Physician-delivered interventions per call**	*n*	1.0 (0.89–1.14)	0.9 (0.6–1.23)	0.90
**Diagnostic interventions**^#^				
*12-lead ECG*	*n* (%)	108 (32.0%)	12 (20.0%)	0.06
*Sonography*	*n* (%)	7 (2.1%)	4 (6.7%)	0.045*
*Blood gas analysis*	*n* (%)	3 (0.9%)	0 (0%)	0.46
**Therapeutic interventions**^#^				
*Symptom-based treatment*	*n* (%)	106 (31.4%)	13 (21.6%)	0.13
*Analgesia*	*n* (%)	63 (18.6%)	12 (20.0%)	0.80
*Oxygen therapy, splinting, and/or positioning*	*n* (%)	23 (6.8%)	6 (10.0%)	0.38
*IV line and crystalloid infusion only*	*n* (%)	14 (4.1%)	4 (6.7%)	0.39
*Shock management*	*n* (%)	12 (3.6%)	4 (6.7%)	0.77
*CPR*	*n* (%)	11 (3.3%)	2 (3.3%)	0.98
*Rapid sequence induction*	*n* (%)	10 (3.0%)	3 (5.0%)	0.41
*Pronouncement of death*	*n* (%)	11 (3.3%)	2 (3.3%)	0.98
*Noninvasive ventilation*	*n* (%)	11 (3.3%)	0 (0%)	0.16
*Fracture/joint reduction*	*n* (%)	5 (1.5%)	2 (3.3%)	0.31
*Invasive arterial pressure monitoring*	*n* (%)	2 (0.6%)	2 (3.3%)	0.05
*Palliative care*	*n* (%)	3 (0.9%)	0 (0%)	0.46
**No intervention delivered**	*n* (%)	47 (13.9%)	7 (11.7%)	0.64
**Calls with interventions only a physician can provide**	*n* (%)	53 (15.7%)	11 (18.3%)	0.61

*CPR* cardiopulmonary resuscitation, *ECG* electrocardiogram, *GEMS* ground emergency medical service, *HEMS* helicopter emergency medical service, *ICU* intensive care unit, *IV* intravenous

^#^ Multiple diagnostic and therapeutic interventions per emergency call could be reported. No obstetric interventions, no pacemaker therapy, no cardioversion, and no emergency surgical interventions (e.g., thoracostomy or chest drain insertion) were reported

* Significant group difference

### Physician characteristics

Physician characteristics did not significantly differ between participating GEMS and HEMS (Table 3).

**Table 3:** Differences in the characteristics of prehospital emergency physicians staffing ground and helicopter emergency medical services

		GEMS	HEMS	*p*-value
*n*		97	23	
Number of physicians per EMS	*n*	22.7 (20.0–25.5)	21.1 (18.0–24.2)	0.73
Female sex of EMS physician	*n (%)*	29 (29.9%)	9 (39.1%)	0.39
Age of EMS physician	*Years*	45 (42.8–47.2)	46.2 (43.1–49.2)	0.86
Specialty status of EMS physician	*n (%)*			0.08
*Specialist*		64 (66.0%)	21 (91.3%)	
*Specialist-in-training*		18 (18.6%)	0 (0%)	
*General practitioner*		14 (14.4%)	2 (8.7%)	
*Missing*		1 (1.0%)	0 (0%)	
Type of specialty of EMS physician (*n* = 103)	*n (%)*			0.89
*Anesthesiology*		61 (74.4%)	15 (71.4%)	
*Internal medicine*		15 (18.5%)	5 (23.8%)	
*Trauma surgery*		2 (2.1%)	0 (0%)	
*Other*		4 (4.9%)	1 (4.8%)	
Experience in prehospital emergency medicine	*Years*	12.4 (10.3–14.5)	15.5 (12.3–18.7)	0.05

*EMS* emergency medical service, *GEMS* ground emergency medical service, *HEMS* helicopter emergency medical service

* Significant group difference

## Discussion

The results of this sub-analysis of a nationwide audit of physician-staffed EMS in Austria indicate that the type, severity, transportation mode, handover place in hospital, and duration of emergency calls differed between GEMS and HEMS. Except for a higher rate of sonography use in HEMS calls, physician-delivered interventions were not different between GEMS and HEMS. Similarly, no differences were found in the characteristics of EMS physicians staffing GEMS and HEMS.

The most important finding of our study was the relevant differences in emergency call profiles between GEMS and HEMS in Austria. The higher median NACA scores in the HEMS group suggest that HEMS are dispatched to emergencies with a higher severity compared to GEMS. Vice versa the rate of over-triage for HEMS was substantially lower than for GEMS implying that emergency control centers have a higher threshold to dispatch HEMS to emergency cases than GEMS. The results of this analysis must, however, be interpreted in light of the fact that the audit took place in early October and thereby before seasonal HEMS had started their operations. Although no published data are available, seasonal HEMS are commonly used as rapid means of transport for patients from skiing resorts to hospitals irrespective of the severity of injury. Therefore, the severity of emergency cases covered by seasonal HEMS might be lower than that reported for HEMS calls in this analysis.

As no reasons for tasking of physician-staffed EMS were collected in the audit, we can only hypothesize on potential factors explaining the high rate of over-triage of physician-staffed EMS, particularly GEMS in this audit. Both suboptimal dispatch criteria and the high density of physician-staffed EMS in Austria appear to be key factors. The fact that only basically trained emergency medical technicians, in over 80% volunteers [2], staff emergency ambulances in Austria may be the reason for the high density of physician-staffed EMS but could also explain the liberal tasking of physician-staffed EMS to low-acuity emergencies. One explanation why a higher rate of over-triage was found for GEMS could be the higher costs, resources and risks associated with HEMS dispatch. Interestingly and in contrast to the published literature, which indicates a particular survival benefit for HEMS use in trauma patients [12–17], we did not find a difference in the medical categories of emergencies to which GEMS and HEMS were tasked. Although trauma cases made up almost 25% of HEMS calls in comparison to 13.3% of GEMS calls, this difference did not reach statistical significance. The relatively low numbers of emergency calls included in the HEMS group could explain this lack of significance.

The proportion of primary calls was lower and the proportion of interfacility transfers higher in HEMS compared to GEMS in our analysis. Although the absolute number of interfacility transfers was only slightly higher in the GEMS than the HEMS group (16 vs. 11), these findings are in line with the current literature reporting that HEMS have a higher proportion of interfacility transfers among their total missions compared to GEMS reflecting the selective use of HEMS for transfers of higher acuity patients and those requiring rapid transport over longer distances [18–20]. Accordingly, a retrospective study analyzing 1784 emergency interfacility transfers to the Innsbruck University Hospital in Austria reported that HEMS was primarily used for longer distances, while GEMS was almost exclusively used for patient transport over distances shorter than 30 km [21]. Correspondingly, the larger operational radius including also remote and mountainous areas together with the higher NACA severity scores in HEMS calls are likely reasons for higher transportation rates of emergency patients attended by HEMS teams. The places in the receiving hospitals where patients were transported to, also differed between GEMS and HEMS. This might once again be the consequence of higher emergency severity scores and higher proportions of interfacility transfers in the HEMS group.

Despite the higher emergency severity of HEMS calls, it was notable that the type and frequency of physician-delivered interventions did not significantly differ between the GEMS and HEMS groups in our analysis. Once again, the low number of HEMS calls and particularly the low number of advanced physician-dependent interventions may have accounted for this observation. Even though the overall rate of prehospital sonography use was very low in our study, ultrasound use was reported more frequently during HEMS calls. This is a likely consequence of the recent initiative of Austria’s largest HEMS provider to equip all helicopters with and train HEMS physician in point-of-care sonography.

Few other studies have so far compared emergency call profiles and interventions between physician-staffed GEMS and HEMS. A register-based, nationwide cohort study from Denmark reported that HEMS was primarily dispatched to patients with severe illness or injury, especially those with time-critical conditions such as cardiovascular emergencies, neurovascular emergencies, and severe trauma. In contrast, GEMS teams were more likely to attend a broader range of emergencies, including those where rapid air transport was not essential or feasible [22]. A retrospective analysis of the German Trauma Register found that patients attended by HEMS were more seriously injured and received more advanced interventions than those attended by GEMS [16]. Studies from Scandinavia and Japan reported similar results [23, 24].

Our study results suggest that physician staffing did not relevantly differ between participating GEMS and HEMS. It revealed an overall predominance of anesthetists practicing as EMS physicians both in GEMS and HEMS. Although the difference did not reach statistical significance, it is noteworthy that HEMS was staffed by specialists in > 90% of cases. With a trend towards a greater prehospital experience, as indicated by more active years as an EMS physician in the HEMS group, the overall experience of EMS physicians staffing GEMS and HEMS in Austria was high.

A key strength of our study is that it included data from all nine federal states of Austria. It, therefore, provides a solid, cross-sectional overview of call profiles, physician-delivered interventions and physician staffing across GEMS and HEMS operations in Austria; however, important limitations need to be considered when interpreting the results of our analysis. First, the fact that data collection occurred only during 24 h led to a small number of emergency calls which could be analyzed. This was particularly the case for the HEMS group and might explain why rare emergencies or certain physician-delivered interventions (e.g., pacemaker therapy, cardioversion, emergency surgical interventions) were not reported in our analysis. Second, the Austrian Emergency Day 2024 audit took place in October. This month was deliberately chosen as it does not fall into the typical tourist seasons in Austria; however, this also implies that call profiles and physician-delivered interventions may be different during winter or summer months. As many tourists in Austria undertake activities in alpine terrains, it is possible that the call profiles and physician-delivered interventions, particularly for HEMS bases, differ during winter and summer months compared to October. Third, determination of the NACA score, especially of potentially life-threatening emergencies (NACA grade 4), may be subject to both individual biases of EMS physicians and billing considerations by HEMS providers. Finally, given the unique staffing of EMS by emergency medical technicians rather than paramedics as well as the high density of physician-staffed EMS in Austria [4, 5, 25], our results might be difficult to extrapolate to other countries.

In conclusion, relevant differences in the call profiles but not physician-delivered interventions or physician staffing exist between GEMS and HEMS in Austria. The HEMS are more frequently tasked to emergencies with a higher severity and conduct interfacility transfers more frequently than GEMS.
